# Additions to the leafhopper genus *Mimotettix* (Hemiptera, Cicadellidae, Deltocephalinae) from Yunnan Province, China

**DOI:** 10.3897/zookeys.786.26402

**Published:** 2018-09-26

**Authors:** Xingtao Wei, Jichun Xing

**Affiliations:** 1 Institute of Entomology, Guizhou University; Special Key Laboratory for Development and Utilization of Insect Resources of Guizhou; Guiyang, Guizhou, 550025, P.R. China Guizhou University Guiyang China

**Keywords:** Homoptera, morphology, taxonomy, distribution, Old World tropics, Scaphoideini

## Abstract

Two new leafhopper species: *Mimotettixmultispinosus***sp. n.** and *M.sinuatus***sp. n.** are described and illustrated from Yunnan, China. A checklist to the species of *Mimotettix* from Yunnan and a key to species from the region are also provided.

## Introduction

The genus *Mimotettix* Matsumura, 1914 (Deltocephalinae: Scaphoideini) is one of the more distinctively marked leafhoppers in the Old World tropics (see Discussion). It was established for a single species, *M.kawamurae*Matsumura 1914, from Taiwan. Later, Kwon and Lee (1979) described another species, *M.curticeps* from South Korea and Webb and Heller (1990) transferred five Indian species to *Mimotettix* from other genera from India. Recently, Li and Xing (2010) described another new species, *M.spinosus*, and made two new combinations: *M.slenderus* (Li & Wang, 2005) and *M.fanjingensis* (Li & Wang, 2005) from China. Meanwhile, Dai et al. (2010) reviewed this genus, based on an examination of the types of most species, and described seven new species and provided a key to the 15 known species. Xing and Li (in Xing et al. 2013) described another new species, *M.articularis* from China, and provided a key to the 10 Chinese species of the genus. Of the latter, three species are distributed in the Palearctic region, i.e., *M.tibetensis* (Tibet) and *M.curticeps* and *M.spinosus* (Gansu, Shaanxi and Henan). Conversely, in Yunnan Province (southern China), one of China’s richest regions in terms of biodiversity, five species are recorded (see Checklist). In the present paper, two new species from Yunnan Province are described which form a separate group from the remaining species of *Mimotettix* based on the structure of the male genitalia (see Discussion) and a key to separate the species from Yunnan is provided. The type specimens of the new species are deposited in the Institute of Entomology, Guizhou University, Guiyang, China (GUGC).

## Material and methods

Male specimens were used for the description and illustration. External morphology was observed under a stereoscopic microscope and characters were measured with an ocular micrometer. Color pictures for adult habitus were obtained by the KEYENCE VHX-1000 system. The genital segments of the examined specimens were macerated in 10% NaOH and drawn from preparations in glycerin jelly using a Leica MZ 12.5 stereomicroscope. Illustrations were scanned with a Canon CanoScan LiDE 200 and imported into Adobe Photoshop CS8 for labeling and plate composition.

The morphological terminology used in the descriptions mainly follows Dai et al. (2010) and Li et al. (2011). Absolute measurements, in millimeters (mm), are used for the body.

## Taxonomy

### Mimotettix

Taxon classificationAnimaliaHemipteraCicadellidae

Matsumura

Mimotettix Matsumura, 1914: 197; Dai et al. 2010: 2; Xing et al. 2013: 4.

#### Type species.

*Mimotettixkawamurae* Matsumura, 1914.

#### Remarks.

For the relationship and diagnosis of *Mimotettix* see Dai et al. (2010: 2)

#### Distribution.

China, Japan and throughout the Old World tropics.

### Checklist of *Mimotettix* species from Yunnan, China

*M.alboguttulatus* (Melichar, 1903)

Distribution: China (Guizhou, Sichuan, Fujian, Guangxi, Yunnan), Japan, India, Sri Lanka, Thailand, Vietnam, Africa.

*M.distiflangentus* Dai, Zhang & Webb, 2010

Distribution: China (Yunnan).

*M.dorsocavatus* Dai, Zhang & Webb, 2010

Distribution: China (Yunnan).

*M.multispinosus* sp. n.

Distribution: China (Yunnan).

*M.robustistylus* Dai, Zhang & Webb, 2010

Distribution: China (Yunnan).

*M.sinuatus* sp. n.

Distribution: China (Yunnan).

*M.spinosus* Li & Xing, 2010

Distribution: China (Guizhou, Yunnan, Shaanxi), Malaysia.

### Key to species (males) of *Mimotettix* from Yunnan Province

**Table:** 

1	Apex of subgenital plate long and thin (Figs 7, 19); connective ‘H’-shaped with distal lateral arms bracing aedeagus (Figs 10, 11, 22); aedeagal process aligned distinctly asymmetrically (Figs 8, 9, 20, 21)	**2**
–	Apex of subgenital plate not long and thin; connective ‘Y’-shaped without distal lateral arms; aedeagal process aligned symmetrically or nearly so	**3**
2	Aedeagal process short, expanded apically with many fine spines (Figs 8, 9)	***M.multispinosus* sp. n.**
–	Aedeagal process elongate, tapered to apex, without spines (Figs 20, 21)	***M.sinuatus* sp. n.**
3	Aedeagal shaft robust, with pair of triangular-shape flanges on dorsal surface	*** M. spinosus ***
–	Aedeagal shaft thin, without pair of triangular-shape flanges on dorsal surface	**4**
4	Aedeagal process with length approximately 1/2 length of shaft	*** M. distiflangentus ***
–	Aedeagal process with length more than 2/3 length of shaft	**5**
5	Aedeagal shaft relatively narrow throughout length in lateral view	*** M. dorsocavatus ***
–	Aedeagal shaft relatively broad throughout length in lateral view	**6**
6	Aedeagal shaft without flanges on dorsal surface; pygophore slightly protruding at ventroposterior angle	*** M. robustistylus ***
–	Aedeagal shaft with narrow flanges on dorsal surface; pygophore acutely rounded posteriorly	*** M. alboguttulatus ***

### Mimotettix alboguttulatus

Taxon classificationAnimaliaHemipteraCicadellidae

(Melichar, 1903)

Thamnotettix alboguttulatus Melichar, 1903: 184–185; synonymised with Mimotettixlateralis (Walker) by Distant 1908: 395, in error.
Paralimnus albomaculatus Distant, 1908: 397; synonymised by Dai et al. 2010: 4, figs 3A–M.
Mimotettix kawamurae Matsumura, 1914: 198, fig. 7; Ishihara 1953: 268, figs 5a–i; synonymised by Dai et al. 2010: 4, figs 3A–M.
Paralimnus lefroyi Distant, 1918: 63; synonymised by Dai et al. 2010: 4, figs 3A–M.
Mimotettix albomaculatus (Distant); Webb and Heller 1990: 7.
Mimotettix lefroyi (Distant); Webb and Heller 1990: 7.
Mimotettix apicalis Li & Wang, 2005: 798, figs 7–12; synonymised by Dai et al. 2010: 4, figs 3A–M.
Mimotettix alboguttulatus (Melichar); Dai et al. 2010: 4, figs 3A–M.

#### Material examined.

4♂♂4♀♀, China: Guizhou Prov., Weining County, 12 August 1977, coll. Plant protection Class 77; 4♂♂7♀♀, Guizhou Prov., Suiyang County, Kuankuoshui, 1 August 1984, coll. Zizhong Li and Lianmin Wang; 2♂♂4♀♀, Guizhou Prov., Fanjing Mt., 12 August 2001, coll. Zizhong Li and Qiongzhang Song; 4♂♂5♀♀, Guizhou Prov., Yanhe County, Mayanghe, 19 May 2007, coll. Yujian Li and Qiongzhang Song; 1♂ (holotype of *Mimotettixapicalis* Li & Wang), Guangxi Autonomous Region, Yuanbaoshan, 13 December 2004, coll. Maofa Yang; 3♂♂7♀♀, Sichuan Prov., Guangyuan City, Shuimogou, 16 August 2007, coll. Jichun Xing; 1♂, Yunan Prov., Xishuangbanna, Menglun, 2 August 2012, coll. Yingjian Wang (GUGC).

#### Distribution.

China (Guizhou, Sichuan, Fujian, Guangxi, Yunnan), Japan, India, Sri Lanka, Thailand, Vietnam, Africa.

### Mimotettix distiflangentus

Taxon classificationAnimaliaHemipteraCicadellidae

Dai, Zhang & Webb, 2010

Mimotettix distiflangentus Dai, Zhang & Webb, 2010: 6, figs 7A–E.

#### Distribution.

China (Yunnan).

### Mimotettix dorsocavatus

Taxon classificationAnimaliaHemipteraCicadellidae

Dai, Zhang & Webb, 2010

Mimotettix dorsocavatus Dai, Zhang & Webb, 2010: 6, figs 8A–F.

#### Distribution.

China (Yunnan).

### Mimotettix multispinosus sp. n.

Taxon classificationAnimaliaHemipteraCicadellidae

http://zoobank.org/FD1E4759-787B-4CC5-86E3-0C046392B952

Figs 1–4 , 5–12

#### Description.

**Figures 1–4.**
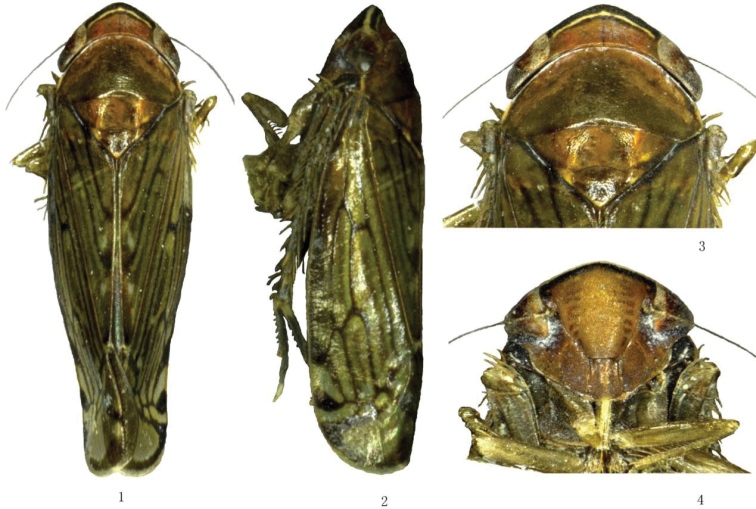
*Mimotettixmultispinosus* sp. n., **1** ♂, dorsal view **2** ♂, lateral view **3** ♂, head and thorax, dorsal view **4** ♂, face, ventral view.

Body reddish brown, vertex with two cream transverse bands anteriorly bordered with dark brown (Figs 1, 3). Eyes black, ocelli pale yellow. Forewings brownish hyaline, with scattered hyaline areas, veins dark brown (Figs 1, 2). Legs dark brown.

Head including eyes slightly wider than pronotum. Vertex roundly produced, slightly shorter medially than the distance between eyes. Ocelli located on anterior margin of vertex, separated from eyes by own diameter. Face slightly flattened, similar in length to width; frontoclypeus narrow, longer than width between eyes; anteclypeus slightly expanded apically (Fig. 4); antennae arising near mid-height of eye in facial view. Pronotum slightly longer than vertex, laterally carinate. Forewings with four apical cells and three subapical cells, outer subapical cell slightly tapered apically, inner subapical cell open basally.

**Figures 5–12.**
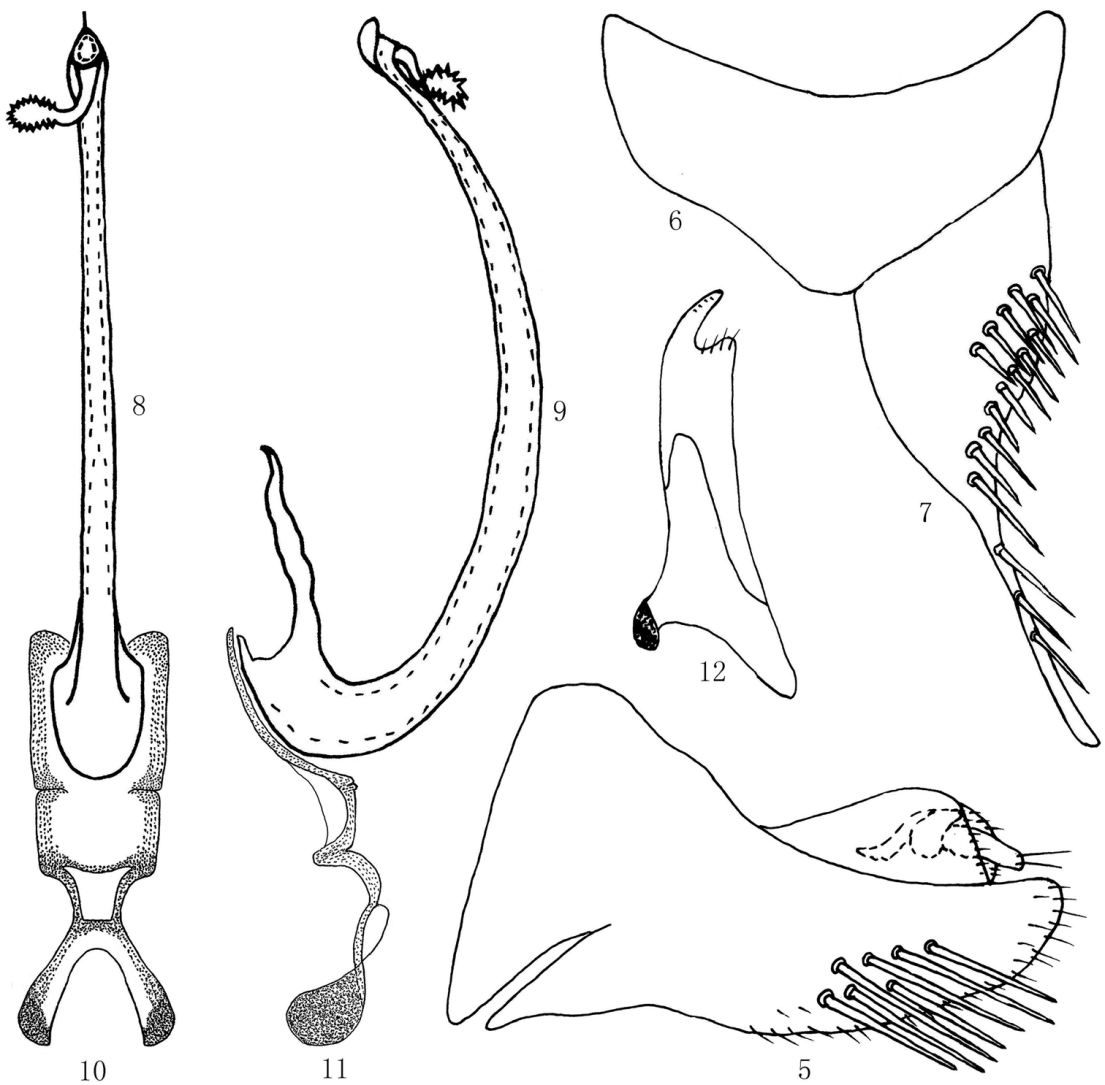
*Mimotettixmultispinosus* sp. n., **5** Pygophore, left lateral view **6** Valve, ventral view **7** Subgenital plate, ventral view **8** Aedeagus, ventral view **9** Aedeagus, lateral view **10** Connective, ventral view **11** Connective, lateral view **12** Style, dorsal view.

*Male genitalia*: Pygofer very elongate and tapered posteriorly in lateral view, with long stout setae on posteroventral margins (Fig. 5). Valve triangulate (Fig. 6). Subgenital plate elongate, tapering posteriorly to lightly sclerotised elongate apex, with uniseriate submarginal row of stout setae ventrolaterally (Fig. 7). Aedeagus with shaft very elongate; apical process relatively short, its length nearly 1/7 length of shaft, curved to one side, with many fine spines; gonopore apical (Figs 8, 9). Connective ‘H’ shaped with arms of stem long and sinuate in lateral view, bracing base of aedeagus (Figs 10, 11). Style relatively narrow, apical process acute, turned laterally (Fig. 12).

#### Measurement.

Length (including tegmen): ♂, 5.5–5.7 mm.

#### Type material.

Holotype ♂, China: Yunnan Prov., Pingbian County, Daweishan, 7 August 2014, coll. Meina Guo (GUGC); paratypes, 2♂♂, same data as holotype except 4 August 2014, coll. Zhengxiang Zhou and 18 August 2017, coll. Yingjian Wang (GUGC).

#### Distribution.

China (Yunnan).

#### Remarks.

The new species is similar to *M.sinuatus***sp. n.**, but can be distinguished by the characters noted in the key. See also Discussion.

#### Etymology.

The new species name is derived from the Latin words “*multi*” and “*spinosus*”, referring to the apical process of aedeagal shaft with many spines.

### Mimotettix robustistylus

Taxon classificationAnimaliaHemipteraCicadellidae

Dai, Zhang & Webb, 2010

Mimotettix robustistylus Dai, Zhang & Webb, 2010: 9, figs 13A-F.

#### Distribution.

China (Yunnan).

### Mimotettix sinuatus sp. n.

Taxon classificationAnimaliaHemipteraCicadellidae

http://zoobank.org/29D47664-D75C-4A1A-85F6-CACE788926FA

Figs 13–16 , 17–23

#### Description.

External features as in *M.multispinosus* (see above), but spots on front wing are lighter. Mesonotum and genae appear to be darker.

*Male genitalia*: As in *M.multispinosus* (see above) but pygofer less elongate (Fig. 17) and aedeagal shaft distinctly broader distally in lateral view, with sinuate elongate apical process tapered to acute apex, half length of shaft (Figs 20, 21). Connective ‘H’ shaped with arms of stem short and not sinuate in lateral view, bracing base of aedeagus (Fig. 22).

#### Measurement.

Length (including tegmen): ♂, 5.4–5.6 mm; ♀, 5.5–5.7 mm.

#### Type material.

Holotype ♂, China: Yunnan Prov., Lvchun County, Huanglianshan, 14 August 2014, coll. Meina Guo (GUGC); paratypes, 1♂2♀♀, same data as holotype except 14 August 2014, coll. Zhengxiang Zhou (GUGC).

#### Distribution.

China (Yunnan).

#### Remarks.

The new species is similar to *M.multispinosus* sp. n. but can be distinguished by the characters noted in the key. See also Discussion.

#### Etymology.

The new species name is derived from the Latin word “*sinuatus*”, referring to the sinuate aedeagal process.

**Figures 13–16.**
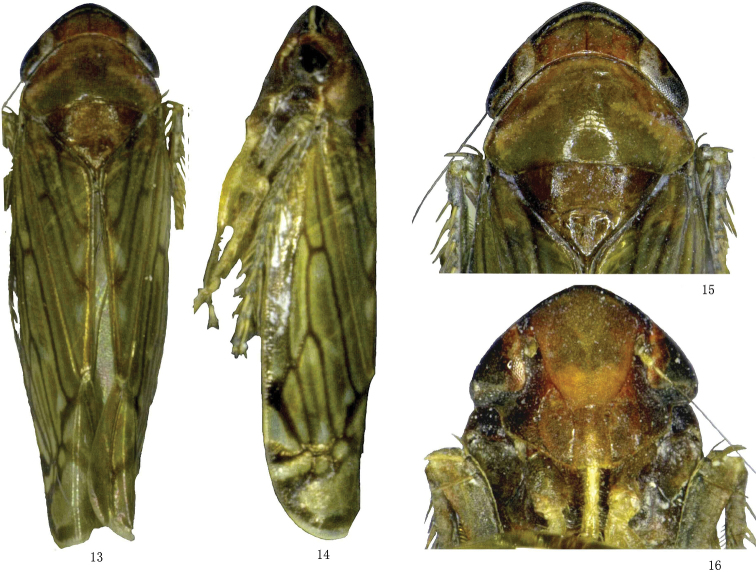
*Mimotettixsinuatus* sp. n., **13** ♂, dorsal view **14** ♂, lateral view **15** ♂, head and thorax, dorsal view **16** ♂, face, ventral view.

**Figures 17–23.**
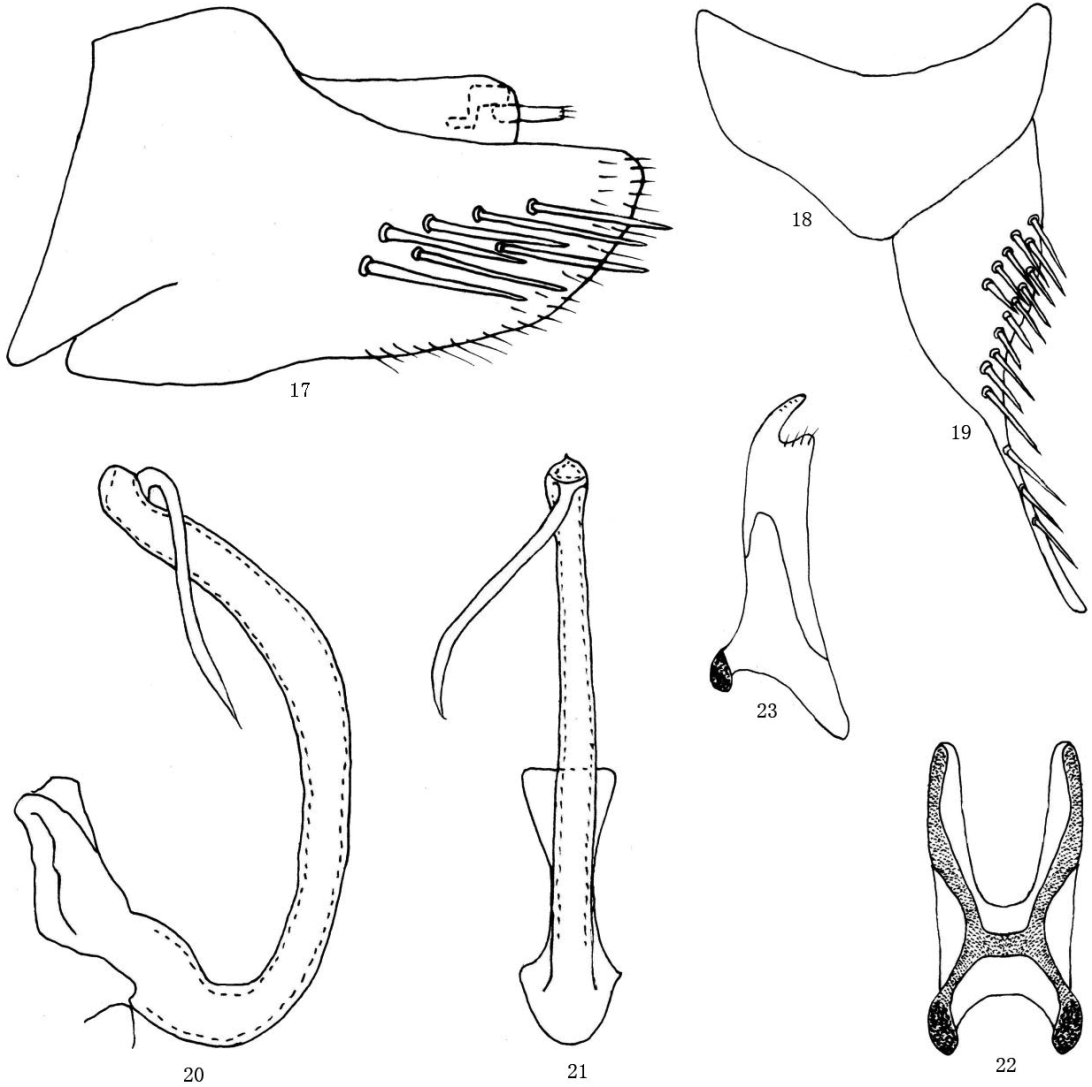
*Mimotettixsinuatus* sp. n., **17** Pygophore, left lateral view **18** Valve, ventral view **19** Subgenital plate, ventral view **20** Aedeagus, lateral view **21** Aedeagus, ventral view **22** Connective, ventral view **23** Style, dorsal view.

### Mimotettix spinosus

Taxon classificationAnimaliaHemipteraCicadellidae

Li & Xing, 2010

Mimotettix spinosus Li & Xing, 2010: 378, figs 1a–g; Dai et al. 2010: 8, figs 12A–F; Li et al. 2011: 135, plates 5–127, figs 1–7.

#### Material examined.

China: 1♂ (Holotype), Guizhou Prov., Libo County, Maolan, 21 October 1998, coll. Zizhong Li (GUGC).

#### Distribution.

China (Guizhou, Yunnan, Shaanxi), Malaysia.

## Discussion

Species of *Mimotettix* are distinctly marked leafhoppers, mainly brown with a series of cream and brown transverse bands on the anterior margin of the head and with hyaline spots on the forewings. In the male genitalia they can be distinguished by the simple aedeagus with the shaft bearing a single apical process directed ventrally. All are very similar in coloration and difficult to distinguish externally, but the structure of the male genitalia is markedly different and separates the genus into two groups: 1) subgenital plate apex extended and very narrow, connective ‘H’ shaped with arms of stem bracing aedeagus, aedeagus with the apical process strongly turned to left or right side of shaft, apex laterally compressed (*M.multispinosus* sp. n. and *M.sinuatus* sp. n.) and, 2) subgenital plate short triangular shaped, connective ‘Y’ shaped, aedeagus with apical process in line with shaft in ventral view or slightly curved to one side, apex not laterally compressed (other species).

## Supplementary Material

XML Treatment for Mimotettix

XML Treatment for Mimotettix alboguttulatus

XML Treatment for Mimotettix distiflangentus

XML Treatment for Mimotettix dorsocavatus

XML Treatment for Mimotettix multispinosus

XML Treatment for Mimotettix robustistylus

XML Treatment for Mimotettix sinuatus

XML Treatment for Mimotettix spinosus
